# Structural Insights into Protein–Aptamer Recognitions Emerged from Experimental and Computational Studies

**DOI:** 10.3390/ijms242216318

**Published:** 2023-11-14

**Authors:** Romualdo Troisi, Nicole Balasco, Ida Autiero, Luigi Vitagliano, Filomena Sica

**Affiliations:** 1 Department of Chemical Sciences, University of Naples Federico II, 80126 Naples, Italy; romualdo.troisi@unina.it; 2 Institute of Biostructures and Bioimaging, CNR, 80131 Naples, Italy; ida.autiero@cnr.it; 3 Institute of Molecular Biology and Pathology, CNR c/o Department of Chemistry, University of Rome Sapienza, 00185 Rome, Italy; nicole.balasco@cnr.it

**Keywords:** aptamer, crystal structure, X-ray crystallography, cryo-electron microscopy, NMR, protein–aptamer interface, molecular dynamics, allostery, ternary complex, protein data bank

## Abstract

Aptamers are synthetic nucleic acids that are developed to target with high affinity and specificity chemical entities ranging from single ions to macromolecules and present a wide range of chemical and physical properties. Their ability to selectively bind proteins has made these compounds very attractive and versatile tools, in both basic and applied sciences, to such an extent that they are considered an appealing alternative to antibodies. Here, by exhaustively surveying the content of the Protein Data Bank (PDB), we review the structural aspects of the protein–aptamer recognition process. As a result of three decades of structural studies, we identified 144 PDB entries containing atomic-level information on protein–aptamer complexes. Interestingly, we found a remarkable increase in the number of determined structures in the last two years as a consequence of the effective application of the cryo-electron microscopy technique to these systems. In the present paper, particular attention is devoted to the articulated architectures that protein–aptamer complexes may exhibit. Moreover, the molecular mechanism of the binding process was analyzed by collecting all available information on the structural transitions that aptamers undergo, from their protein-unbound to the protein-bound state. The contribution of computational approaches in this area is also highlighted.

## 1. Introduction

Intermolecular interactions represent key events in all biological processes. In living organisms, partnerships between biomolecules are characterized by high specificities and a wide range of binding affinities [1]. Proteins, key factors in all biochemical pathways, are promiscuous biomolecules whose activities generally rely on intricate partnerships that they establish with many different chemical entities ranging from individual atoms/ions to huge macromolecules. In this scenario, it is not surprising that the modulation of protein partnerships including those established with other proteins represents a remarkable option in investigations aimed at developing new biomolecules of diagnostic and/or therapeutic interest [2,3,4]. However, the interactions that proteins form with large biomolecules usually involve huge interfaces that cannot be efficiently inhibited with small molecules [5]. Therefore, it has been traditionally believed that antibodies could represent the obvious solution to this issue [6]. However, the discovery that DNA- or RNA-based polynucleotides endowed with the ability to specifically target proteins, including those not involved in interactions with nucleic acids in physio–pathological conditions, could be developed with reasonable costs has changed this perspective. Indeed, a wide range of proteins can be targeted by nucleic acids, denoted as aptamers, with affinities and specificities comparable to those exhibited by antibodies [7,8,9,10,11]. Aptamers are typically generated by using a procedure denoted as Systematic Evolution of Ligands by EXponential Enrichment (SELEX) in which randomly generated libraries of DNA or RNA sequences presenting all possible bases in each position are exposed to the target [12,13,14,15,16,17]. Afterward, the sequences that do recognize and bind the target are eluted and amplified by PCR and used in subsequent steps of selection of the strongest binders. Since the set-up of the SELEX approach three decades ago [18,19], thousands of different aptamers directed against proteins of therapeutic and/or diagnostic interest have been developed [10,14,20,21,22,23]. The many distinctive properties of aptamers compared to protein-based therapeutics, which include the possibility of setting flexible designs, their rather straightforward production, and the opportunity to easily modify them, have generated a remarkable enthusiasm for their potential to become effective biomarkers or drugs [7,24]. Although many aptamers have become tools of extreme importance in basic science [22,25,26,27,28,29,30,31], for many years only a single aptamer-based drug was in the marketplace, i.e., pegaptanib sodium (Macugen by Pfizer/Eyetech) that was approved in 2004 by the FDA for macular degeneration [32]. Very recently, the FDA approved a second RNA aptamer, i.e., avacincaptad pegol (Izervay by Iveric Bio/Astellas) for geographic atrophy secondary to age-related macular degeneration [33]. Although this success may represent a turning point in the perception of aptamers as attractive potential drugs, also considering their potential for treating acute conditions such as thrombolysis and cytokine release syndrome [33], there is a clear gap between the expectations and initial enthusiasm and the real outcome of so many investigations and trials [34]. There are of course many possible explanations for this so-called aptamer paradox [35]. Among others, the limited information currently available on aptamer structure and their mechanism of action represents a significant factor [34]. Indeed, despite the thousands of aptamers generated and characterized, a small fraction of them have been structurally investigated. Although the first structural characterization of a protein–aptamer complex was reported nearly three decades ago [36], limited progress has been made over the years. Initial analyses on protein–aptamer complexes were reported by Van der Oost and coworkers in 2012 [37]. In 2016, a study of the structural themes that characterize protein–aptamer recognition described the only 16 protein–aptamer complexes available at that time [38]. More recently, forty-five structures of these complexes were surveyed by Novoseltseva et al. [39], while some selected examples were analyzed by Ge Zhang and coworkers in 2021 [40]. Here, by exhaustively exploring the entire content of the Protein Data Bank (PDB), which contains more than 200,000 protein structures, we identified 144 PDB entries containing atomic-level information on protein–aptamer complexes. Interestingly, a remarkable increase in the number of determined structures was observed in the last two years as a consequence of the effective application of the cryo-electron microscopy (cryo-EM) technique to these systems. The intricate architecture and the versatile stoichiometry of protein–aptamer complexes were analyzed. Moreover, the molecular mechanism of the binding process was analyzed by collecting all available information, including that retrieved from computational studies, on the structural transitions that aptamers undergo from their protein-unbound to the protein-bound state.

## 2. Protein–Aptamer Complexes in the Protein Data Bank: Identification and Classification

### 2.1. Procedure Used to Select the Structures of Aptamers and Their Complexes with Proteins and Classification Tools

An initial ensemble of structures containing aptamers was generated by interrogating the RCSB PDB (release of 31 July 2023) using the term “aptamer” as a query in the search box of the website (https://www.rcsb.org/ accessed on 9 November 2023). This search led to the identification of 435 PDB entries potentially containing three-dimensional structures of aptamers. The list of the PDB codes is reported in Table S1. Since aptamers are promiscuous biomolecules that are frequently developed to bind different types of ions and molecules, a heterogeneous ensemble of complexes in addition to aptamers in their ligand-free states was produced by this preliminary search. To identify protein–aptamer complexes, the search was further refined by considering only the entries also containing the term “Protein” in the “Polymer Entity Type” category. The application of these criteria led to the selection of 176 entries. Each of these was manually inspected to include only entries that effectively contained protein/peptide–aptamer complexes. Moreover, for the sake of completeness, the entries 8F3C, 8G00, 8G1S, 8G2W, 8G4W, and 8G7E, which were missed in the initial search but strictly related to 8G8Z, were added to the ensemble. This procedure led to the identification of 146 structures of protein–aptamer complexes. Among them, eight entries appeared to contain ternary complexes in which two aptamers simultaneously bind to different regions of the same protein molecule. Accordingly, in ternary complexes, two distinct protein–aptamer interfaces are present. Therefore, our search led to the identification of 154 protein–aptamer interfaces. The chemical classification of the aptamers involved in these complexes indicated a significant variability, as we detected 84 DNA, 65 RNA, 3 DNA/RNA hybrid (NA-hybrid), and 2 peptide aptamers. Although peptide aptamers are generally obtained by using protocols that present intriguing analogies with the SELEX procedure commonly used to generate DNA and RNA aptamers [41], since this review was focused on the interactions underlying the formation of complexes between proteins/peptides and nucleic acids, they were not further considered (PDB IDs 6TBT and 7EZW). Entries reporting riboswitch structures that contain an aptamer domain were also included in the dataset.

The final ensemble of the 144 PDB entries containing 152 protein–aptamer interfaces, along with some details (experimental technique, resolution, and release date), is reported in Table S2. Each entry was assigned a number (from #1 to 144) that will be used throughout the text.

The interface area (IA) and the number of direct (not mediated by water molecules) hydrogen bonds (H-bonds) present at the protein–aptamer interfaces of each structure of the dataset were computed with the PISA program [42] available online (https://www.ebi.ac.uk/pdbe/pisa/, accessed on 9 November 2023) applying the default parameters and settings. Linear regression analyses were performed to gain insights into the relationships between parameters (IA, number of protein–aptamer H-bonds, and aptamer length in terms of number of nucleotides). The significance of the correlation coefficients (R-values) is expressed with the *p*-value. Table 1 reports data describing the partners in the complexes and their interactions.

### 2.2. Chronological Evolution of Aptamer Structures Reported in the PDB: Impact of the Different Methodologies

The first structural characterization of a protein–aptamer complex [36] dates back to the early 1990s with the determination of the structure of the complex between human α-thrombin, the key enzyme of the coagulation cascade [120], and a 15-mer antiparallel G-quadruplex DNA aptamer (known as TBA). This latter exerts a strong anticoagulant activity by interacting with the thrombin fibrinogen-binding site (exosite I) [121]. Indeed, the first structures of this complex (entries #1–3, PDB ID 1HUT, 1HAO, and 1HAP) were released in the PDB between 1994 and 1996 (Table S2). In detail, the first structure of the thrombin–TBA complex (entry #1, PDB ID 1HUT) was solved by X-ray crystallography at 2.90 Å resolution [36]. Although the central core of TBA was defined, the poor quality of the electron density in the flexible regions of the aptamer did not clarify the disposition of the loops in relation to the grooves. In 1996, the structure of the complex was re-determined with better diffraction data (2.80 Å resolution) and by using in the fitting procedure two models of the aptamer differing for the relative orientation of TBA and thrombin [43]. The refined structures seemed equally correct. In particular, in one model (entry #3, PDB ID 1HAP), TBA interacts with thrombin exosite I by its TGT loop, whereas in the other (entry #2, PDB ID 1HAO), it interacts by its TT loops. This ambiguity was finally resolved only in 2011–2012 when the crystal structures of the complexes between thrombin and either modified or unmodified TBA (entries #27, 35, and 36, PDB IDs 3QLP, 4DIH, and 4DII) provided new and more accurate structural information on the thrombin–aptamer recognition process [60,64]. In particular, the new structures definitively pointed out that the aptamers interact with thrombin exosite I by their TT loops, a feature in line with model 1HAO (entry #2). It is worth noting that, over the years, several new TBA variants, containing a 3′-3′/5′-5′ inversion of polarity site, additional terminal moieties, or modifications at the level of the nucleobases, the sugar portions, and/or the phosphodiester linkages, were developed [122]. In many cases, the interactions of these TBA analogs with thrombin were also studied by X-ray crystallography (entries #49, 50, 60, 76, 77, 99–101, and 128–131, PDB IDs 4LZ1, 4LZ4, 5CMX, 6EO6, 6EO7, 6Z8V, 6Z8W, 6Z8X, 7ZKL, 7ZKM, 7ZKN, and 7ZKO) [72,80,90,104,114], obtaining some structural clues for their improved or weaker performances. Moreover, the X-ray structures of new-selected and highly effective thrombin-binding DNA aptamers, such as NU172 and M08s-1, were recently solved (entries #87, 88, and 135, PDB IDs 6EVV, 6GN7, and 8BW5) [96,117]. Overall, these observations clearly indicate that thrombin has a prototypical role in allowing the understanding of the basis of protein–aptamer recognition [121]. Its special role will be also highlighted in the analysis of ternary complexes simultaneously involving a single protein and two aptamers (see below) [82,107,114].

**Figure 1:**
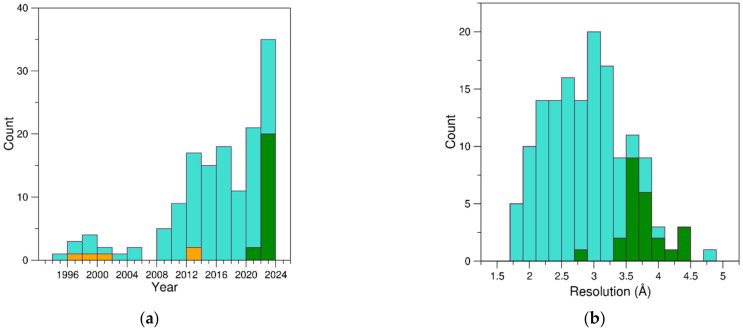
Distribution of (**a**) the 144 PDB entries of protein–aptamer complexes reported since 1994 and (**b**) their resolution. Bars are colored according to the experimental technique used to determine the structure: X-ray crystallography (cyan), cryo-EM (green), and solution NMR (orange).

However, a global analysis of the structural data that emerged in the last three decades indicates that a rather limited number of successful structural characterizations were reported up to 2008 (Figure 1). A significant increment in the structures of these complexes was observed in the last decade, with an average number of yearly deposited structures of ~eight. A remarkable increment is evident in the last two years, with 35 structures reported since 2022, which represents 24% of all protein–aptamer complexes. All major structural biology techniques that can provide atomic-level models of biological macromolecules have been successfully applied in this field. Indeed, of the 144 entries, 117 (~81%) were determined by X-ray crystallography, 22 (~15%) by cryo-EM, and 5 (~4%) by solution NMR. The analysis in Figure 1a indicates a non-homogeneous chronological distribution of these studies. Indeed, while X-ray crystallography has been constantly used over the years, the NMR studies were concentrated at the turn of the millennium (1997–2000) and in 2013. On the other hand, the huge methodological and technological advances in cryo-EM, which is revolutionizing the entire field of structural biology, are heavily affecting the characterization of protein–aptamer recognition. Indeed, although the first cryo-EM structures of a protein–aptamer complex were reported only in 2021 (entries #108 and 109, PDB ID 7OZW and 7P15) [108], in the last two-year period (2022–2023), the number of structures determined by using this technique has overcome that solved by X-ray crystallography (20 *versus* 15). This observation suggests that the structural biology of protein–aptamer complexes is anticipating trends that are expected to occur in a few years in the entire field. Indeed, while in the period January 2022–July 2023 the total number of structures solved by X-ray crystallography deposited in the PDB almost doubled those determined by cryo-EM (10,329 *versus* 5376), it is commonly believed that this scenario will be reversed soon [123,124].

The analysis of the main features of the structures determined by using these different methodologies reflects their intrinsic specificities. Indeed, structures investigated by X-ray crystallography generally present higher resolution and better accuracy than those determined by cryo-EM (Figure 1b). This is particularly evident when structures solved in the same period (2022–2023) are compared (Figure S1a). Although there is no major variation in the aptamer size (Figure S1b), which is essentially dictated by the set-up of the SELEX experiment, cryo-EM provided structural information on aptamer targeting larger proteins (Figure S1c).

### 2.3. Characterization of the Interfaces That Stabilize Protein–Aptamer Complexes

**Figure 2:**
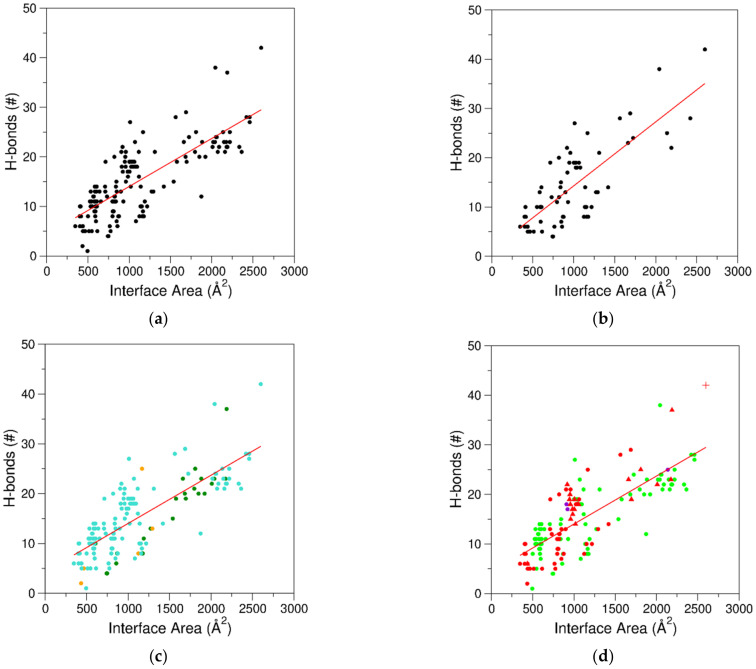
Correlation between interface areas and the number of H-bonding interactions for (**a**) the entire dataset of 152 protein–aptamer interfaces (R = 0.78, *p* < 10^−5^) and (**b**) the non-redundant ensemble including 67 interfaces (R = 0.78, *p* < 10^−5^). The entire dataset is colored (**c**) according to the experimental technique used to determine the structures (cyan for X-ray crystallography, green for cryo-EM, and orange for NMR) and (**d**) according to the chemical nature of the aptamer (green for DNA, red for RNA, and purple for NA-hybrid). In (**d**), complexes containing riboswitch aptamers and the complex of tRNA^Gln^ var-AGGU are represented with up-pointing triangle and plus symbols, respectively.

As reported in Figure 2, protein–aptamer interfaces may present rather different sizes (in the range of 340–2600 Å^2^). In line with a previous analysis conducted on a significantly smaller database [39], a remarkable correlation between intermolecular protein–aptamer H-bonds, as defined by PISA [42] (see above), and the buried area was observed. The linear regression analysis demonstrated the high significance of this correlation (R-value 0.78 and *p*-value < 10^−5^) (Figure 2a). The correlation was also evident when a further selection to the ensemble of structures was applied by removing those reporting complexes formed by the same protein with aptamers presenting minor modifications that did not significantly affect the value of the buried surface. In these cases, only the highest resolution entry was considered. In this non-redundant dataset (67 entries reported in bold in Table 1), the R-value was still 0.78 (*p*-value < 10^−5^) (Figure 2b). It must be underlined that H-bonds are not the only interactions that drive the binding of aptamers to the target proteins. The paradigmatic example of the binding between the abovementioned TBA and its variants to the electropositive region of thrombin (exosite I) indicates that other forces, such as electrostatic, hydrophobic, π-π stacking, and cation-π interactions, are exploited in this partnership. Indeed, while a thymine of a TT loop occupies a hydrophobic crevice delineated on the thrombin surface by the side chains of a tyrosine and two isoleucine residues, the nucleobase of another thymine in another TT loop forms a π-π stacking interaction with the side chain of a second tyrosine [64,104,114]. Concurrently, the remaining two thymines form a cyclic arrangement with two arginine residues that stack on the guanines of the first G-tetrad of the aptamer and generate two cation-π/H-bond stair motifs [82].

In general, the different protein–aptamer interactions inspired numerous analytical detection assays, each based on the regulation of a specific binding force [125].

**Figure 3:**
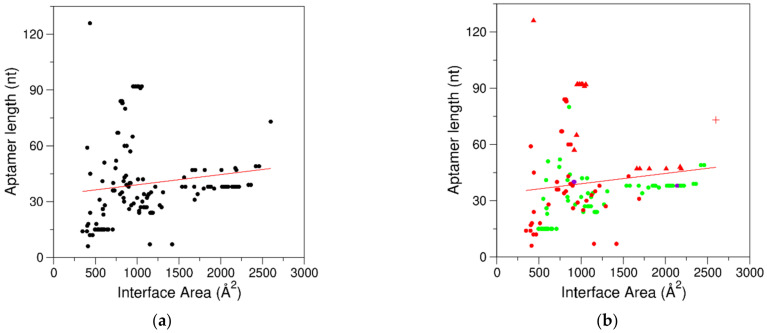
Correlation between interface areas and aptamer lengths for (**a**) the entire dataset of 152 interfaces (R = 0.14, *p* = 0.09). (**b**) Data are colored according to the chemical nature of the aptamer (green for DNA, red for RNA, and purple for NA-hybrid). In (**b**), complexes containing riboswitch aptamers and the complex of tRNA^Gln^ var-AGGU are represented with up-pointing triangle and plus symbols, respectively.

From the methodological point of view, as expected, NMR has been used to characterize complexes with interfaces of low–medium sizes. Complexes with the largest buried areas have been investigated by either X-ray crystallography or cryo-EM (Figure 2c). The analysis of the interfaces as a function of the aptamer type (RNA, DNA, and NA-hybrid) indicated that, in the framework of large variabilities, the smallest buried areas (IA < 490 Å^2^) were observed for RNA aptamers (Figure 2d). On the other hand, the dimension of the interface presented little correlation with the aptamer size, as small interfaces were observed for aptamers of very different sizes (Figure 3a). This observation is valid independently of the aptamer chemical nature (Figure 3b). This finding indicates that aptamers may present a relevant structural complexity beyond the motif that directly anchors the protein partner. Indeed, significant regions essential for their folding may not be involved in the partnership.

**Figure 4:**
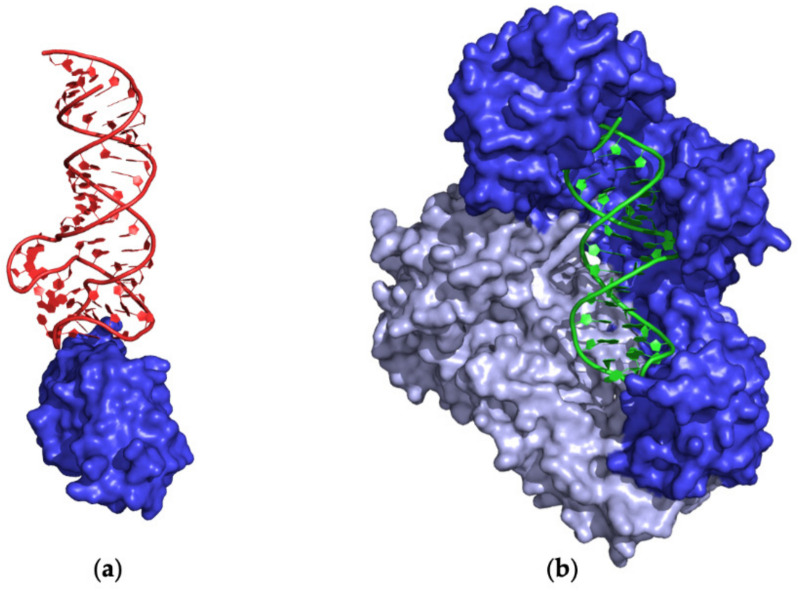
Selected examples of PDB structures endowed with a (**a**) small (entry #43, PDB ID 4M4O) and (**b**) large (entry #59, PDB ID 5D3G) protein–aptamer interface. (**a**) Lysozyme C and (**b**) HIV-1 reverse transcriptase are shown in blue. DNA and RNA aptamers are shown in green and red, respectively. Different shades of blue are used for the different protein chains of HIV-1 reverse transcriptase.

Notably, most of the structures endowed with the smallest IAs are formed by either short RNA aptamers (< 25 nucleotides) or small proteins (< 100 amino acid residues). These include the small aptamers involved in interactions with viral capsid subunits: (i) F6, F5, F7, and F5/2AP10, targeting the bacteriophage MS2 coat protein (entries #5–7 and 12, PDB IDs 6MSF, 5MSF, 7MSF, and 1U1Y) [45,46,51]; (ii) the aptamer bound to the genome polyprotein HPeV-1 (entry #72, PDB ID 5MJV) [87]. A small IA is also observed in the structure of the small anti-Fc aptamer bound to the Fc fragment of IgG1 (entry #19, PDB ID 3AGV) [57] and the SAM-I riboswitch targeting the small RNA-binding protein YbxF (entry #34, PDB ID 3V7E) [63]. In the NMR structures (entries #39 and 40, PDB IDs 2RSK and 2RU7) of aptamer R12 bound to the P16 peptide from the major prion protein [66,67], both partners are characterized by a reduced size, leading to small IAs. Finally, despite the large sizes of both partners, complexes of aptamers minE (59 nucleotides) and minF (45 nucleotides) with lysozyme C (129 residues) (entries #43 and 44, PDB ID 4M4O and 4M6D) [70] are among the structures with the smallest interface areas (Figure 4a). In particular, only ~400 Å^2^ of the solvent-accessible surface of the protein is buried upon the binding of the aptamers. Moreover, the limited contacts between the protein and the phosphate backbone determine an unusually small fraction (~18%) of electrostatic interaction in the lysozyme–aptamer interface [70].

The largest IA is present in the structure of the complex of aptamer tRNA^Gln^ var-AGGU bound to glutaminyl-tRNA synthetase (entry #9, PDB ID 1EXD) [48]. Large areas (IA > 2000 Å^2^) are exhibited by two classes of aptamers: (i) DNA aptamers interacting with HIV-1 reverse transcriptase subunits (entries #59, 64–68, 80–83, 105–107, 110–114, and 126, PDB IDs 5D3G, 5HLF, 5HP1, 5HRO, 5I3U, 5I42, 5XN0, 5XN1, 5XN2, 6BHJ, 7OXQ, 7OZ2, 7OZ5, 7LRI, 7LRM, 7LRX, 7LRY, 7LSK, and 7Z2G) [79,83,84,92,93,108,109] (Figure 4b); (ii) RNA aptamers (riboswitches) targeting DNA-directed RNA polymerase subunits (entries #140–142, PDB IDs 8G4W, 8G7E, and 8G8Z) [118]. In all these structures, the aptamer is involved in interactions with more than one protein subunit. Among the largest IAs is also the complex of a DNA aptamer with the DUX4 protein (entry #94, PDB ID 6U82) [100]. It is worth noting that large IAs are usually correlated with a sort of protein wrapping around the aptamer structure.

Interestingly, the analysis of the protein residue types that are present at the protein–aptamer interface in selected and representative structures (Figure S2) is indicative of well-defined general trends. The residue that occurs with the highest frequency is arginine. This finding clearly highlights the role of electrostatic interactions in the stabilization of these interfaces. This observation is corroborated by the significant presence of other positively charged residues such as lysine and histidine. A high frequency was also detected for the aromatic residues (tyrosine, tryptophan, phenylalanine), in line with the role that π-π stacking and cation-π interactions may play in this partnership (see above). Similar trends were reported in a statistical analysis of the interaction between proteins and nucleic acids [126].

### 2.4. Stoichiometry of Protein–Aptamer Complexes

#### 2.4.1. Monomeric Proteins

**Figure 5:**
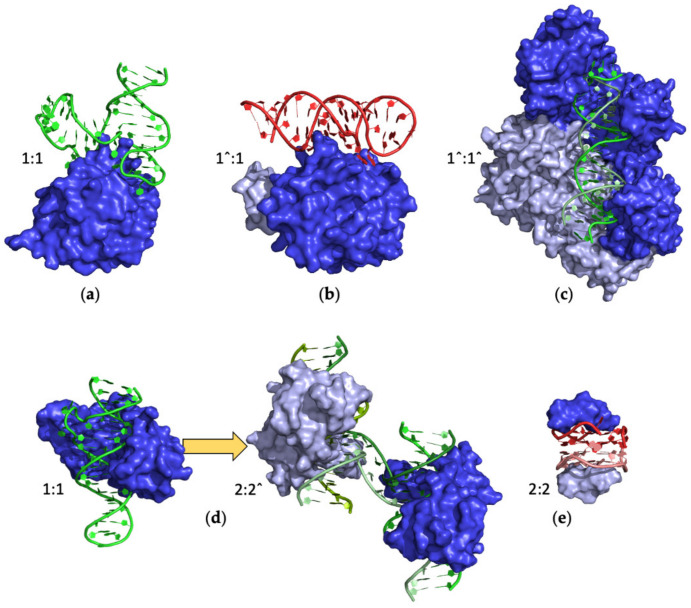
Selected examples of PDB structures of protein–aptamer complexes formed by monomeric proteins (blue): (**a**) von Willebrand factor (entry #16, PDB ID 3HXO), (**b**) coagulation factor Xa (entry #78, PDB ID 5VOE), (**c**) HIV-1 reverse transcriptase (entry #106, PDB ID 7OZ2), (**d**) double homeobox protein 4 (from left to right: entries #94 and 93, PDB ID 6U82 and 6U81), and (**e**) P16 peptide from a major prion protein (entry #40, PDB ID 2RU7). DNA and RNA aptamers are indicated in green and red, respectively. Different shades of the same color are used for the different protein/aptamer chains. For each structure, the protein–aptamer stoichiometry is indicated.

Most of the complexes contain a single protein and aptamer chain (protein/aptamer ratio 1:1) (Table 1). This class includes also proteins consisting of a light and a heavy chain derived from a single-chain polypeptide precursor such as thrombin, coagulation factor Xa, HIV-1 reverse transcriptase, and BL3-6 and bevacizumab Fabs. For these latter, the notation 1ˆ:1 was used (Table 1). Analogously, for entries #105–107 (PDB IDs 7OXQ, 7OZ2, and 7OZ5) [108], the notation 1ˆ:1ˆ was used, as the aptamer bound to HIV-1 reverse transcriptase consists of two distinct chains forming a double helix. Illustrations of selected examples of complexes 1:1, 1ˆ:1, and 1ˆ: 1ˆ are reported in Figure 5a–c.

A special case in this class is represented by the aptamers bound to the DUX4 protein (entries #93 and 94, PDB IDs 6U81 and 6U82) [100]. In detail, while #94 reports a canonical 1:1 complex formed by a single-chain DNA aptamer forming a hairpin interspersed with a bulge loop, #93 is endowed with a more articulated stoichiometry (2:2ˆ). Indeed, it reports an aptamer variant formed by two DNA strands. However, the presence of a non-complementary region (bulge residues) leads to the formation of a Holliday junction structure in which two strands are swapped between two complexes (Figure 5d) [100].

The NMR structures (entries #39 and 40, PDB IDs 2RSK and 2RU7) [66,67] of the R12 aptamer bound to the P16 peptide from a major prion protein represent another example of two interacting aptamers, each bound to a peptide chain (2:2). The two G-quadruplex aptamers stack up on each other while binding P16 peptides on the free faces (Figure 5e).

Two distant monomers of the same protein (interleukin-6) are linked by an aptamer chain in entry #52 (PDB ID 4NI9) [73] eventually leading to the formation of a 2:2 complex. Since a sTable 1:1 complex was reported for this system (entry #51, PDB ID 4NI7) [73], the interaction of SL1025 aptamer with the second protein chain is not essential and is likely generated by the crystal packing.

##### Ternary Complexes

Monomeric proteins may be simultaneously targeted by two aptamers, thus forming ternary complexes (1ˆ:2 or 1:2 in Table 1). In particular, eight structures of ternary complexes involving either thrombin (entries #62, 63, 104, and 129–131 PDB IDs 5EW1, 5EW2, 7NTU, 7ZKM, 7ZKN, and 7ZKO) [82,107,114] or the spike protein S1 from SARS-CoV-2 (entries #143 and 144, PDB IDs 8J1Q and 8J26) [119] have been reported until now.

**Figure 6:**
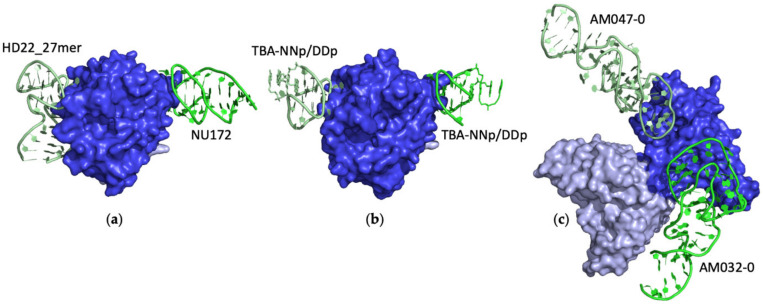
Selected examples of PDB structures of protein–aptamer ternary complexes. (**a**) Thrombin interacts with NU172 at exosite I and HD22_27mer at exosite II (entry #104, PDB ID 7NTU). (**b**) Two molecules of TBA-NNp/DDp bind the two thrombin exosites (entry #129, PDB ID 7ZKM). (**c**) RBD of SARS-CoV-2 spike protein S1 interacts with AM032-0 at the ACE2-binding site and AM047-0 at a distal site (entry #143, PDB ID 8J1Q). Different shades of the same color are used for the different protein/aptamer chains. In (**c**), the Fab domain of the imdevimab antibody is in light blue.

In addition to the previously mentioned (Section 2.2) exosite I, the thrombin surface hosts a second larger electropositive region, named exosite II, that is targeted by several thrombin natural interactors, such as heparin [121]. This exosite is also the binding site of two different classes of either RNA (Toggle) or DNA (HD22) aptamers that display unique structural features, as emerged from the structural characterization of their complexes (entries #14, 42, and 61, PDB IDs 3DD2, 4I7Y, and 5DO4) [53,69,81]. In the last decades, mutual through-bond effects between the two exosites in the presence of various thrombin binders were pointed out by biochemical and biophysical studies [107,127,128,129,130,131,132,133,134,135,136,137]. To structurally reveal the allosteric effects among thrombin exosites induced by the simultaneous binding of specific aptamers, ternary complexes in which a single thrombin molecule is sandwiched between two distinct aptamers bound to the two protein exosites were elucidated [82,107]. In detail, the structures of the complexes of thrombin with HD22_27mer at exosite II and either TBA variants (TBA∆T3 or TBA∆T12, entries #62 and *63*, PDB IDs 5EW1 and 5EW2) [82] or NU172 (#104, PDB ID 7NTU) [107] (Figure 6a) at exosite I were reported. Some of these structural studies showed that small conformational changes occur at exosite II in the ternary complexes compared to the thrombin–HD22_27mer binary complex [82]. Interesting details were derived by applying computational techniques [107,138]. Indeed, molecular dynamics (MD) studies led to the classification of the long-range inter-exosite communication in thrombin as dynamic allostery. According to this model, the allosteric regulation is related to an aptamer-guided dynamic transmission of the structural information from one exosite to the other that, however, does not encounter marked conformational rearrangements [107,138].

Recently, a TBA variant (named TBA-NNp/DDp), in which the 3′ and 5′ ends were, respectively, conjugated with two electron-rich (1,5-dialkoxy naphthalene) and two electron-deficient (1,8,4,5-naphthalenetetra-carboxylic diimide) moieties, was studied [114]. The X-ray structures of the complex between thrombin and TBA-NNp/DDp (entries #129–131, PDB IDs 7ZKM, 7ZKN, and 7ZKO) [114], released in the PDB in 2022, unexpectedly showed the simultaneous binding of two molecules of the same aptamer at the two distinct exosites (Figure 6b). The overall structural analysis of this variant, corroborated by solution studies, revealed that the peculiar ability of this aptamer to interact, in addition to exosite I, also with a secondary low-affinity binding site on exosite II, is strictly related to the solvophobic behavior of the terminal modifications. It must be underlined that in one case (entry #131, PDB ID 7ZKO), the crystal was formed by an equimolar mixture of 1ˆ:1 and 1ˆ:2 protein–aptamer complexes [114]. Interestingly, the analysis of the interacting surface of TBA and thrombin at exosite II, which is non-canonical for this aptamer, indicated that it is similar to the interface area of TBA at exosite I and different from the surfaces commonly detected in the complexes with HD22_27mer and Toggle-25t aptamers targeting exosite II with high affinity (Figure S3).

Finally, in the last year, the cryo-EM structures of two 1:2 ternary complexes (entries #143 and 144, PDB IDs 8J1Q and 8J26) [119] in which the receptor-binding domain (RBD) of SARS-CoV-2 spike protein S1 is simultaneously bound to two different aptamers, AM032-0 and AM047-0, or their derivatives, at two distinct binding sites, were reported (Figure 6c). These studies showed that the binding of angiotensin-converting enzyme 2 (ACE2), the viral receptor protein of the host cell, to the spike protein S1 is hindered by the AM032 aptamer family. This aptamer is indeed able to competitively block the ACE2-binding site in the protein RBD domain. On the other hand, the AM047 aptamers allosterically inhibit ACE2 by binding to a completely distinct region in the spike. It has to be noted that in these structures, the protein is also bound to the Fab domain of the imdevimab antibody used to increase the overall size of the complexes thus making them suitable for cryo-EM studies. These cryo-EM structures represented the starting models to design a bivalent aptamer that would strongly inhibit SARS-CoV-2 pseudovirus infection [119].

#### 2.4.2. Homomeric Proteins

**Figure 7:**
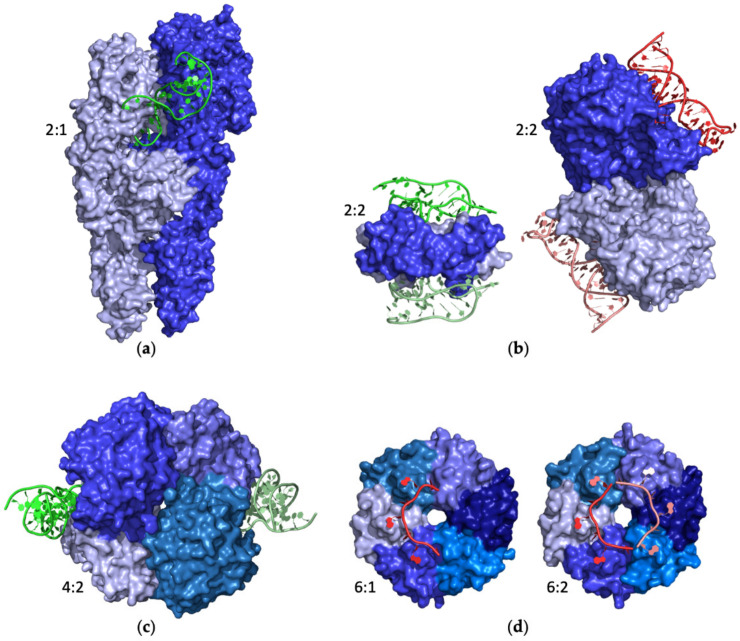
Selected examples of PDB structures of protein–aptamer complexes formed by homomeric proteins (blue): (**a**) insulin receptor (entry #118, PDB ID 7YQ3), (**b**) nerve growth factor (on the left, entry #58, PDB ID 4ZBN) and glutamate carboxypeptidase 2 (on the right, entry #89, PDB ID 6RTI), (**c**) lactate dehydrogenase (entry #70, PDB ID 5HRU), and (**d**) RNA-binding protein Hfq (from left to right: entries #20 and 21, PDB ID 3HSB and 3AHU). DNA and RNA aptamers are shown in green and red, respectively. Different shades of the same color are used for the different protein/aptamer chains. For each structure, the protein–aptamer stoichiometry is indicated.

Several structures of protein–aptamer complexes involving homomeric proteins have been reported over the years. Homodimeric proteins were shown to interact with either one (protein–aptamer 2:1) or two (2:2) aptamers. Both chains of the homodimer are simultaneously involved in the interaction with a single aptamer molecule in the structures of (i) K1 and K2 aptamers bound to the Tet repressor protein (entries #90 and 91, PDB IDs 6SY4 and 6SY6) [98], (ii) A43 and A62 aptamers bound to the insulin receptor (entries #118–120, PDB IDs 7YQ3, 7YQ4, and 7YQ5) [112] (Figure 7a). For this latter system, the structure of a 2:2 complex in which each A62 aptamer interacts with both protein chains of the homodimer was also reported (entry #121, PDB ID 7YQ6) [112]. This is also the case for the complexes containing aptamers (i) SL5 and SL4 bound to the platelet-derived growth factor B (entries #37 and 38, PDB IDs 4HQU and 4HQX) [65], (ii) SL1049 bound to nerve growth factor (entry #58, PDB ID 4ZBN) [78] (Figure 7b).

On the other hand, structures of 2:2 complexes in which each aptamer targets only one chain of the homodimer involve (i) tJBA8.1 aptamer bound to transferrin receptor protein 1 (entry #132, PDB ID 7ZQS) [115], (ii) anti-Fc aptamer bound to the Fc fragment of IgG1 (entry #19, PDB ID 3AGV) [57], (iii) A9g aptamer bound to glutamate carboxypeptidase 2 (entry #89, PDB ID 6RTI) [97] (Figure 7b).

For higher-order homomeric proteins, the following structures were reported [58,68,86,99]. The homo-tetramer lactate dehydrogenase is targeted by aptamers pL1 (entries #70 and 71, PDB ID 5HRU and 5HTO) [86] (Figure 7c), 2008s (entry #41, PDB ID 3ZH2) [68], and cubamer (entry #92, PDB ID 6TXR) [99] that interact with two out of its four chains (4:2). Finally, two structures of the homohexameric RNA-binding protein Hfq interacting with either one (6:1) or two (6:2) AGr aptamer chains were solved (entries #20 and 21, PDB ID 3HSB and 3AHU) [58] (Figure 7d).

A comparison of the redundant protein–aptamer interfaces, which are present in the same biological assembly, in complexes 2:2, 4:2, and 6:2 indicated that the interfaces are essentially preserved (Figure S4).

#### 2.4.3. Large Protein Assemblies

Aptamers have been developed also to target large assemblies [139,140]. A global survey of the structural studies performed on these complexes indicated that they may be classified into two distinct groups.

**Figure 8:**
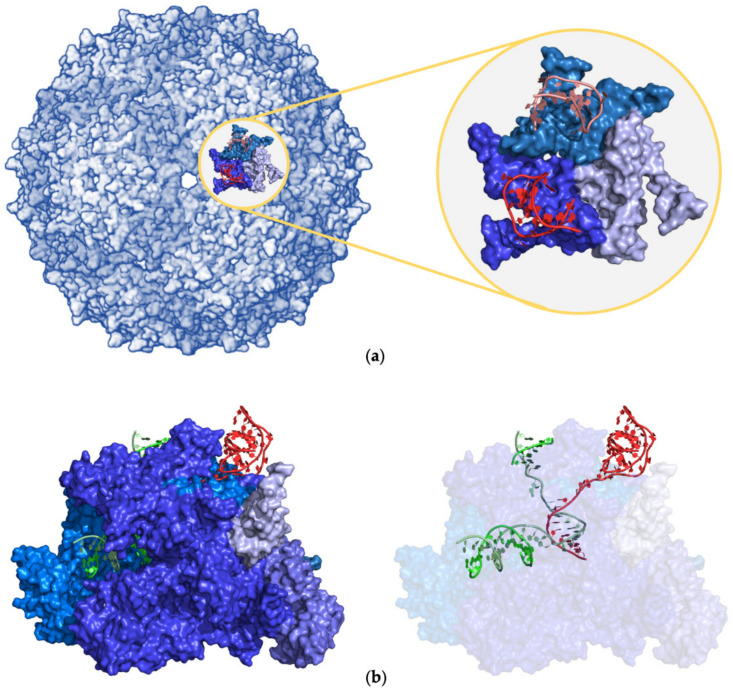
Selected examples of PDB structures of protein–aptamer complexes in large protein assemblies: (**a**) MS2 coat protein (entry #6, PDB ID 5MSF) and (**b**) DNA-directed RNA polymerase (entry #136, PDB ID 8F3C). Proteins and RNA aptamers are shown in blue and red, respectively. DNA in (**b**) is shown in green. Different shades of the same color are used for the different protein/nucleic acid chains.

The first class comprises aptamers binding viral capsid subunits. Two distinct viruses have been so far targeted and structurally characterized. In particular, three studies were focused on the bacteriophage MS2 that was targeted by the RNA aptamers F5, F5/2AP10, F6, and F7 (entries #5–7 and 12, PSB IDs 6MSF, 5MSF, 7MSF, and 1U1Y) [45,46,51]. All these variants interact and specifically bind a single capsid protein (Uniprot ID P03612) (Figure 8a). To this class also belongs the complex formed by an RNA aptamer and the genome polyprotein of the human parechovirus 1 (HPeV-1) (entry #72, PDB ID 5MJV) [87]. In this case, the aptamer binds two (VP1 and VP3) of the three capsid protein subunits, forming the construct that was structurally characterized. As anticipated above (Section 2.3), despite the large size of the targeted assembly, these complexes present very limited interface areas.

A completely distinct example is represented by RNA aptamers targeting RNA polymerase proteins. This group was characterized in two structural studies [52,118]. The first large complex of this class was structurally characterized in 2005 at low resolution (3.80 Å) by X-ray crystallography (entry #13, PDB ID 2B63) [52]. In this structure, the FC* aptamer interacts with 2 out of the 12 subunits that compose the construct of DNA-directed RNA polymerase II. Conversely, the recent cryo-EM characterization of the complex between the aptamer module of an RNA riboswitch and the multichain DNA-directed RNA polymerase demonstrated that the aptamer interacts with the beta and the beta’ subunits of the protein (entries #136–142, PDB IDs 8F3C, 8G00, 8G1S, 8G2W, 8G4W, 8G7E, and 8G8Z) [118]. In addition to these interactions, the aptamer is also in contact with the DNA bound to the polymerase, making a mixed DNA–RNA duplex (Figure 8b). These additional interactions increase the IA to values >2500 Å^2^. The global interface formed by this aptamer with the protein/DNA assembly is one of the largest present in the PDB.

## 3. Structural Versatility of Aptamers Targeting Proteins: Insights into the Recognition Mechanism

Molecular recognition is a complex process whose quantitative interpretation requires a deep understanding of the many factors involved. The knowledge of the interactions formed in adducts or complexes is *per se* not sufficient for understanding the physico–chemical basis of the recognition process. Indeed, the characterization of the intrinsic structural/dynamic properties of the interacting partners is an important step for the elucidation of this process. Indeed, although, for the protein partner, minor global structural modifications are expected and detected, aptamers may undergo significant structural rearrangements upon complex formation. Unfortunately, due to the intrinsic flexibility of aptamers, their characterizations in ligand-free states are not frequent. To comprehensively address this issue, in addition to the dataset of structures of protein–aptamer complexes, we also interrogated the PDB, looking for aptamers that were not bound to proteins. This was carried out by selecting in the PDB the aptamer structures for which the “Polymer” keyword only contained the “Nucleic Acid” expression. Using this approach, we retrieved an ensemble of 291 entries. The sequences of the aptamers of this dataset were systematically compared with those present in the dataset of the complexes. The results of this comprehensive comparison are reported in Tables S3 and S4.

In the case of DNA aptamers, several protein-bound aptamers were shown to share significant sequence similarities with those present in the ensemble of the protein-unbound ones (Table S3). In most cases, the protein-bound states share high similarities with at least one counterpart of the protein-unbound ensemble, with root-mean-square deviations (RMSD) lower than 2 Å. This observation indicates that the binding of these aptamers to the protein target has marginal effects on their structure. This observation is not surprising, since DNA aptamers tend to adopt rather rigid structural motifs, such as duplexes or G-quadruplexes. A significant exception to this trend was detected when the comparison was performed between protein-unbound TBA and the early structures of the aptamer complexed to thrombin (entries #1 and 3, PDB IDs 1HUT and 1HAP) [36,43]. It is important to note that, as reported above (Section 2.2), the modeling of the structure of this aptamer was a controversial issue [36,43,60,64].

For RNA aptamers, although protein-bound and -unbound aptamers frequently share highly similar fragments, the occurrence of the same aptamer in the two ensembles is a quite rare event (Table S4). Using as thresholds for the shared sequence in each pair (protein-bound and protein-unbound) at least 85% of sequence identity and 55% of sequence coverage, we identified three pairs that corresponded to the aptamers interacting with (i) the 30S ribosomal protein S8 (entry #53, PDB ID 4PDB) [74], (ii) the transcription factor NF-κB (entry #11, PDB ID 1OOA) [50], (iii) the Fab BL3-6 (entries #133 and 134, PDB IDs 8D29 and 8DK7) [116]. The similarity of the protein-bound/-unbound aptamer pairs was confirmed by the inspection of the related literature, in which comparative analyses of the components of each pair were reported [74,116,141]. In addition to these pairs, we also compared the structures of the R12 aptamer, which binds the P16 peptide of a prion protein (entries #39 and 40, PDB IDs 2RSK and 2RU7) [66,67], with that of its analog R12-A-R12 (in which two R12 aptamers are tandemly connected) characterized in its protein-unbound state [142].

**Figure 9:**
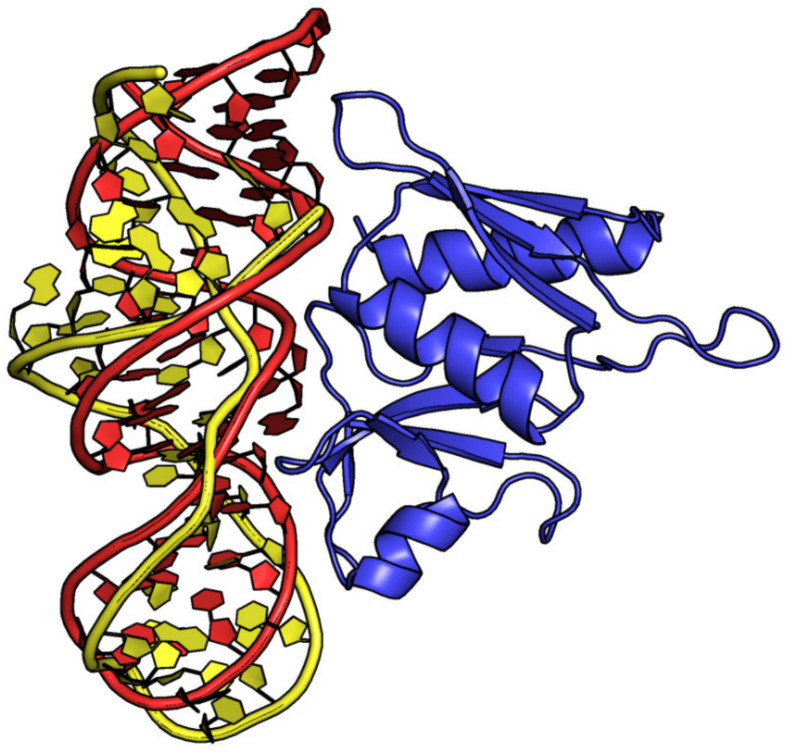
Structural superposition of the RNA aptamer targeting the ribosomal protein S8 (blue) in the protein-unbound (yellow, PDB ID 2LUN) and -bound (red, entry #53, PDB ID 4PDB) states.

**Figure 10:**
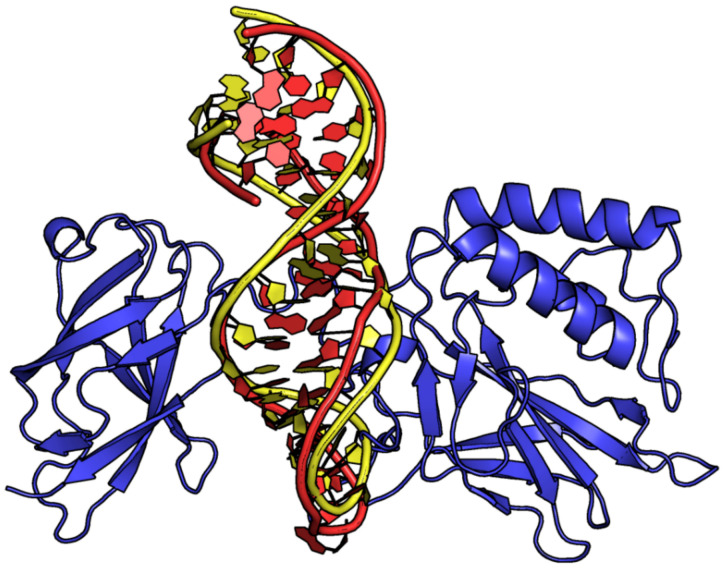
Structural superposition of the RNA aptamer targeting NF-κB (blue) in the protein-unbound (yellow, PDB ID 2JWV) and -bound (red, entry #11, PDB ID 1OOA) states.

**Figure 11:**
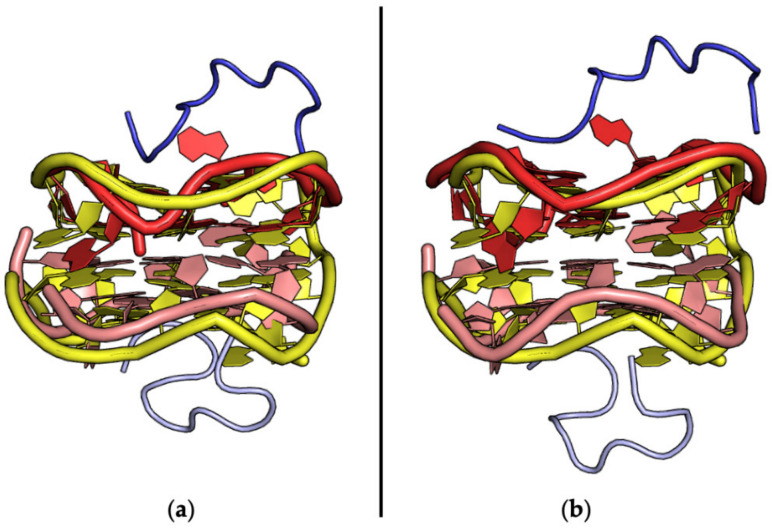
Structural superposition of the anti-prion RNA aptamer in the protein-unbound state (yellow, PDB ID 6K84) and bound to the P16 peptide from a major prion protein (blue), as reported in (**a**) entry #39 (red, PDB ID 2RSK) and (**b**) entry #40 (red, PDB ID 2RU7). Different shades of the same color are used for the different protein/aptamer chains.

**Figure 12:**
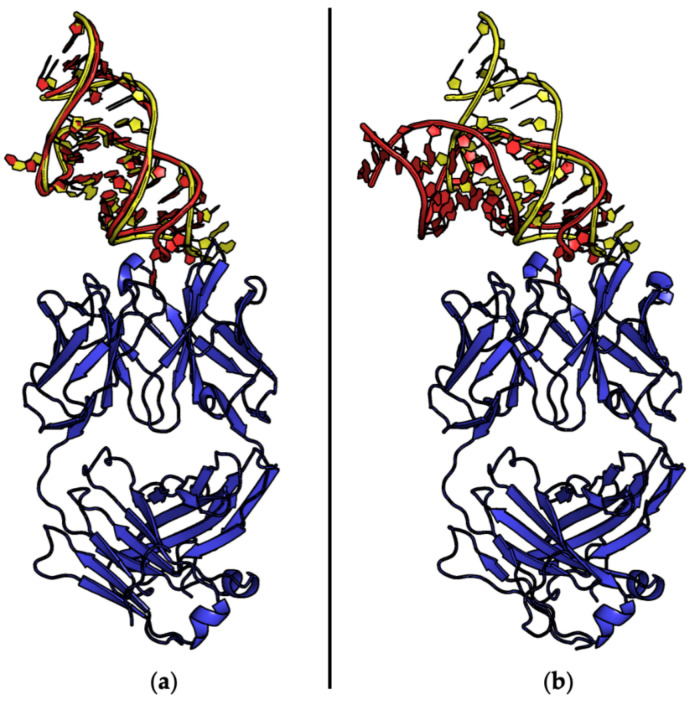
Structural superposition of the theophylline RNA aptamer in the protein-unbound state (yellow, PDB ID 1O15) and bound to Fab BL3-6 (blue), as reported in (**a**) entry #134 (red, PDB ID 8DK7) and (**b**) entry #133 (red, PDB ID 8D29). The protein-bound aptamer in (**b**) does not bind theophylline.

A global analysis of these four cases was suggestive of different responses of these aptamers to protein binding (Figure 9, Figure 10, Figure 11 and Figure 12). Indeed, as detailed below, significant alterations in the aptamer structures were detected for those targeting the ribosomal protein S8 and the transcription factor NF-κB. On the other hand, the R12 structure exhibited minor rearrangements upon the binding to the prion protein motif. Even smaller structural perturbations were caused by the binding of the theophylline aptamer to the Fab BL3-6.

In more detail, the binding of the aptamer to the protein S8 was associated with remarkable changes in the RNA structure that led to a novel combination of nucleobase interactions [74] (Figure 9). The change in topology observed in the aptamer is indicative of the remarkable plasticity of its structure and of the role of the protein target in dictating structural variations. These observations were suggestive of an induced-fit mechanism in the recognition process. An extensive MD simulation study, which was performed on both the complex and the protein-unbound aptamer, supported this conclusion by showing that the protein-bound conformation was not present in the ensemble of states adopted by its protein-unbound form [143].

As for the protein S8, the targeting of NF-κB was also associated with remarkable changes in the aptamer structure. In particular, the comparison of the protein-unbound structure of this aptamer [141], which was determined by NMR, with that found in the crystallographic structure of the complex [50] highlighted remarkable variations in the tetraloop and the internal loop regions (Figure 10). In this case, however, replica exchange MD simulations, which provide an enhanced sampling of the conformations adopted by biomolecules, indicated that some bound-like states of the aptamer were present in the conformational space of the protein-unbound form [144]. This finding is suggestive of a population selection mechanism in protein–aptamer recognition. Collectively, MD investigations carried out on aptamers targeting proteins showed that the recognition may occur through distinctive binding mechanisms such as induced fit or population selection.

For the pairs (bound/unbound states) characterized by limited variations of the aptamer structure upon target recognition, such as in the case of the prion peptide (Figure 11), this may be ascribed to the small size of the peptide target and/or to the rigid G-quadruplex structure adopted by the aptamer.

On the other hand, Fab–aptamer interactions deserve a specific description. The intrinsic flexibility of aptamer structures makes the experimental characterizations of their ligand-free state extremely complicated. One of the most ingenious strategies developed to overcome this problem was proposed by Piccirilli and coworkers [145]. It consists of the addition of a motif, which is specifically recognized and bound with high affinity by an ad hoc designed Fab, to the modular sequence of RNA aptamers. Therefore, it is not surprising that the Fab-binding version of the aptamer targeting the small molecule theophylline does not undergo major modifications upon Fab association (Figure 12a). Notably, the structure of this aptamer is remarkably different in the absence of theophylline [116] (Figure 12b). In this context, it is important to note that the binding of another aptamer (DIR2) to its Fab cognate may have important consequences on its long-range dynamics [144].

## 4. Conclusions and Perspectives

The comprehensive and systematic analysis of the entire structural content of the PDB here reported revealed that the number of protein–aptamer complexes has been rapidly increasing in the last couple of years. This was essentially due to the possibility of studying these complexes by taking advantage of the impressive methodological and technical advances of the cryo-EM methodology. If this trend continues in the next years, a remarkable increase in the structural information related to these systems is expected to be available shortly. Indeed, the possibility of tackling the non-trivial structural characterization of these complexes with two complementary methodological options opens new scenarios in the field. Depending on the investigated system and the structural details needed, the optimal choice could be made by considering that cryo-EM can easily be applied to large complexes and, therefore, to big protein targets, while X-ray crystallography generally provides higher-resolution models.

The analysis of the extent of the protein–aptamer interfaces, in line with a recent independent report [39], indicated that they are correlated with the number of intermolecular H-bonds formed. Although a large variability of the buried surface area was observed, the values found were similar to those detected in generic protein–nucleic acid complexes [126]. It is also interesting to note that the areas of the protein–aptamer interfaces are qualitatively similar to those of the protein–protein interfaces [146].

Although a large variability of the buried surface area was observed, the values found are similar to those detected in generic protein–RNA complexes. The present survey also indicated that the protein–aptamer complexes that have been structurally characterized have different stoichiometry and, sometimes, articulated architectures. Indeed, although monomeric proteins are still the most represented class of proteins in our dataset, the number of oligomeric proteins is progressively increasing (Table 1). In recent years, a growth of data available for ternary complexes was detected. Indeed, the structure of novel complexes between thrombin and two aptamers anchored to the two protein exosites was reported [82,107]. These structural characterizations coupled with extensive MD studies provided interesting insights into the allosteric effects caused by the binding to one exosite on the other [107,138]. The exosite II binding mode of TBA, an aptamer traditionally studied for its ability to inhibit protein exosite I, was unraveled [114]. The analysis of this non-canonical binding, integrated within a comparative analysis of the interacting surfaces detected in aptamer binary and ternary complexes formed by thrombin, indicated that the aptamer type, rather than the exosite area, dictates the size of the buried area upon complex formation.

If experimental structural data related to protein–aptamer complexes have been growing in the last years, the characterization of the structural properties of protein-targeting aptamers in their unbound state is still very poor. Although this type of information is essential for the definition of the protein–aptamer recognition process, only a few of these aptamers have been characterized in their protein-unbound state. Nevertheless, depending on the system, the formation of the complex may have either a remarkable or a marginal impact on the protein structure. However, considering the intrinsic flexibility of aptamers that often prevents their structural characterization in the free state, it is likely that protein–aptamer recognition is associated with a significant alteration of the nucleic acid structure. The data here collected on the few aptamers structurally characterized in both the bound and the unbound states suggest that modifications induced by the binding are more likely for RNA rather than for DNA aptamers.

Taking into account the wealth of information provided by computational studies performed in this field [147,148], this gap of information could be filled by systematically investigating the intrinsic structural/dynamics in the unbound state of the aptamers whose structure complexed with proteins is available. Studies of this type conducted on the aptamers targeting the ribosomal S8 protein [143] or NF-κB [144] demonstrated that protein–aptamer recognition may rely on different mechanisms, i.e., induced fit or population selection.

In addition to molecular modeling and MD simulations, the recent success of machine learning approaches in the prediction of both protein structures and protein complexes raises the question of whether RNA/DNA folding and the structures of protein–nucleic acid complexes will be accurately predicted in the next few years [149,150]. However, the limited structural content of the PDB concerning protein–RNA complexes compared to that regarding individual proteins or protein–protein complexes makes the development of machine-based approaches more difficult in this context [151]. Nevertheless, the prediction of protein–aptamer interactions [152] will be promoted significantly by deep learning methods taking advantage of the information provided by AlphaFold on the protein side [153,154] and by exploiting the growing ensemble of protein–nucleic acid structures.

## Figures and Tables

**Table 1:** Ensemble of 144 PDB entries of complexes containing 152 protein–aptamer interfaces. The 67 entries of the non-redundant dataset (see Section 2.3) are in bold. The protein–aptamer stoichiometry refers to the biological assembly. Aptamer length is expressed as the number of nucleotides (nt).

Entry	PDB ID	Aptamer		Interacting Protein or Peptide	Protein–Aptamer Stoichiometry	Interface Area (Å^2^)/H-Bonds	Ref.
		Name	Type/ Length (nt)				
1	1HUT	TBA	DNA/15	Thrombin (exosite I)	1ˆ:1	500.6/8	[36]
2	1HAO				1ˆ:1	546.6/5	[43]
3	1HAP				1ˆ:1	494.6/1	
4	**1ULL**	35-mer RRE RNA aptamer I	RNA/35	HIV-1 Rev peptide	1:1	1167.3/25	[44]
5	**6MSF**	F6	RNA/14	MS2 coat protein	Large assembly	346.8/6	[45]
6	**5MSF**	F5	RNA/18		Large assembly	416.2/8	[46]
7	**7MSF**	F7	RNA/14		Large assembly	400.0/8	
8	**484D**	27-mer RNA aptamer II	RNA/27	HIV-1 Rev peptide	1:1	1293.1/13	[47]
9	**1EXD**	tRNA^Gln^ var-AGGU	RNA/73	Glutaminyl-tRNA synthetase	1:1	2599.3/42	[48]
10	**1EXY**	33-mer RNA aptamer	RNA/33	HTLV-1 Rex peptide	1:1	1124.7/8	[49]
11	**1OOA**	29-nt RNA aptamer	RNA/29	Nuclear factor NF-κB	2:2	958.9/21	[50]
12	1U1Y	F5/2AP10	RNA ^§^/17	MS2 coat protein	Large assembly	403.2/10	[51]
13	**2B63**	FC*	RNA ^§^/31	RNA polymerase II	Large assembly	1688.1/29	[52]
14	3DD2	Toggle-25t	RNA ^§^/26	Thrombin (exosite II)	1ˆ:1	904.6/21	[53]
15	**3EGZ**	Tetracycline aptamer (riboswitch)	RNA/65	U1 small nuclear ribonucleoprotein A	1:1	946.3/19	[54]
16	**3HXO**	ARC1172	DNA/42	Von Willebrand factor	1:1	1011.1/27	[55]
17	3HXQ				1:1	1070.8/23	
18	3IRW	Vc2 (riboswitch)	RNA ^§^/92	U1 small nuclear ribonucleoprotein A	1:1	952.4/20	[56]
19	**3AGV**	anti-Fc	RNA ^§^/24	Fc fragment of IgG1	2:2	438.5/6	[57]
20	**3HSB**	AGr	RNA/7	RNA-binding protein Hfq	6:1	1419.3/14	[58]
21	**3AHU**				6:2	1152.8/10	
22	3MUM	G20A mutant Vc2 (riboswitch)	RNA ^§^/92	U1 small nuclear ribonucleoprotein A	1:1	957.0/18	[59]
23	3MUR	C92U mutant Vc2 (riboswitch)	RNA ^§^/92		1:1	1006.7/19	
24	3MUT	G20A/C92U mutant Vc2 (riboswitch)	RNA ^§^/92		1:1	981.2/17	
25	3MUV				1:1	1007.7/19	
26	3MXH	Vc2 (riboswitch)	RNA ^§^/92		1:1	984.8/16	
27	3QLP	mTBA	DNA/15	Thrombin (exosite I)	1ˆ:1	656.6/11	[60]
28	3UCU	Vc2 (riboswitch)	RNA ^§^/92	U1 small nuclear ribonucleoprotein A	1:1	1055.9/18	[61]
29	3UCZ				1:1	1004.6/17	
30	3UD3	C92U mutant Vc2 (riboswitch)	RNA ^§^/92		1:1	960.0/15	
31	3UD4				1:1	1018.6/14	
32	**3UZS**	C13.28	RNA/28	G-protein coupled receptor kinase 2	1:1	613.5/5	[62]
33	**3UZT**	C13.18	RNA/18		1:1	512.3/5	
34	**3V7E**	SAM-I (riboswitch)	RNA/126	RNA-binding protein YbxF	1:1	435.3/6	[63]
35	4DIH	TBA	DNA/15	Thrombin (exosite I)	1ˆ:1	565.0/12	[64]
36	4DII				1ˆ:1	542.7/11	
37	**4HQU**	SL5	DNA ^§^/24	Platelet-derived growth factor B	2:2	1158.3/8	[65]
38	4HQX	SL4	DNA ^§^/24		2:2	1166.5/9	
39	2RSK	R12	RNA/12	P16 peptide from major prion protein	2:2	434.2/2	[66]
40	**2RU7**				2:2	466.7/5	[67]
41	**3ZH2**	2008s	DNA/35	Lactate dehydrogenase	4:2	1310.3/21	[68]
42	**4I7Y**	HD22_27mer	DNA/27	Thrombin (exosite II)	1ˆ:1	1079.5/18	[69]
43	**4M4O**	minE	RNA/59	Lysozyme C	1:1	403.1/6	[70]
44	**4M6D**	minF	RNA/45		1:1	439.2/5	
45	4KZD	Spinach	RNA ^§^/84	Fab BL3-6	1ˆ:1	820.5/11	[71]
46	4KZE				1ˆ:1	815.3/11	
47	4Q9Q				1ˆ:1	801.3/9	
48	4Q9R				1ˆ:1	811.2/9	
49	4LZ1	TBA∆T12	DNA ^§^/15	Thrombin (exosite I)	1ˆ:1	533.8/13	[72]
50	4LZ4	TBA∆T3	DNA ^§^/15		1ˆ:1	540.6/11	
51	**4NI7**	SL1025	DNA ^§^/32	Interleukin-6	1:1	1000.8/19	[73]
52	4NI9				1:1	1118.2/16	
53	**4PDB**	RNA-2	RNA/38	30s ribosomal protein S8	1:1	898.6/13	[74]
54	**4R8I**	NOX-E36	RNA ^§^/40	Chemokine CCL2	1:1	714.2/19	[75]
55	**4WB2**	NOX-D20	NA-hybrid ^§^/40	Complement anaphylatoxin C5a	1:1	922.3/17	[76]
56	4WB3				1:1	910.0/18	
57	**4YB1**	G20A mutant Vc2 (riboswitch)	RNA/91	U1 small nuclear ribonucleoprotein A	1:1	1039.9/18	[77]
58	**4ZBN**	SL1049	DNA ^§^/28	Nerve growth factor	2:2	930.2/11	[78]
59	**5D3G**	38NT2,4-methyl	DNA ^§^/38	HIV-1 reverse transcriptase	1ˆ:1	2189.9/22	[79]
60	**5CMX**	RE31	DNA/31	Thrombin (exosite I)	1ˆ:1	551.7/10	[80]
61	**5DO4**	Toggle-25t/AF113-18	RNA ^§^/25	Thrombin (exosite II)	1ˆ:1	1024.6/18	[81]
62	5EW1	TBA∆T3	DNA ^§^/15	Thrombin (exosite I)	1ˆ:2	523.7/11	[82]
		HD22_27mer	DNA/27	Thrombin (exosite II)		1116.2/21	
63	5EW2	TBA∆T12	DNA ^§^/15	Thrombin (exosite I)	1ˆ:2	533.6/10	
		HD22_27mer	DNA/27	Thrombin (exosite II)		1044.2/19	
64	5HLF	38NT2,4-methyl	DNA ^§^/38	HIV-1 reverse transcriptase	1ˆ:1	2218.7/23	[83]
65	5HP1		DNA ^§^/39		1ˆ:1	2333.4/22	[84]
66	5HRO		DNA ^§^/38		1ˆ:1	2149.5/23	
67	5I3U	38NT2,4-methyl (variant)	DNA ^§^/39		1ˆ:1	2362.5/21	
68	5I42	38NT2,4-methyl	DNA ^§^/38		1ˆ:1	2051.9/24	
69	**5HRT**	RB011	DNA ^§^/34	Autotaxin ENPP2	1:1	1139.7/14	[85]
70	**5HRU**	pL1	DNA/34	Lactate dehydrogenase	4:2	1136.8/10	[86]
71	5HTO				4:2	1081.5/7	
72	**5MJV**	-	RNA/6	Genome polyprotein HPeV-1	Large assembly	412.0/10	[87]
73	**5UC6**	SL1067	DNA ^§^/23	Interleukin-1α	1:1	597.1/7	[88]
74	**6B14**	Spinach	RNA/83	Fab BL3-6	1ˆ:1	821.2/12	[89]
75	6B3K				1ˆ:1	826.1/12	
76	6EO6	TBA-T4W	DNA ^§^/15	Thrombin (exosite I)	1ˆ:1	707.6/14	[90]
77	6EO7	TBA-T4K	DNA ^§^/15		1ˆ:1	640.6/13	
78	**5VOE**	11F7t	RNA ^§^/36	Coagulation factor Xa	1ˆ:1	730.9/12	[91]
79	5VOF				1ˆ:1	710.2/13	
80	5XN0	38NT2,4-methyl (variant)	DNA ^§^/38	HIV-1 reverse transcriptase	1ˆ:1	2073.1/21	[92]
81	5XN1				1ˆ:1	2061.2/24	
82	5XN2				1ˆ:1	2017.1/23	
83	**6BHJ**	38NT2,4-methyl (variant)	NA-hybrid/ 38		1ˆ:1	2137.1/25	[93]
84	**6CF2**	35-mer RRE RNA aptamer I	RNA/35	HIV-1 Rev protein	1:1	819.4/20	[94]
85	**6DB8**	DIR2s	RNA/60	Fab BL3-6	1ˆ:1	876.4/8	[95]
86	6DB9				1ˆ:1	849.1/13	
87	**6EVV**	NU172	DNA/26	Thrombin (exosite I)	1ˆ:1	589.8/10	[96]
88	6GN7				1ˆ:1	588.6/8	
89	**6RTI**	A9g	RNA ^§^/43	Glutamate carboxypeptidase 2	2:2	1563.0/28	[97]
90	**6SY4**	K1	RNA/43	Tet repressor protein	2:1	848.3/7	[98]
91	**6SY6**	K2	RNA/39		2:1	873.0/8	
92	**6TXR**	Cubamer	RNA ^§^/38	Lactate dehydrogenase	4:2	1218.1/10	[99]
93	**6U81** ^‡^	-	DNA/ (17 + 20)_2_	Double homeobox protein 4 DUX4	2:2ˆ	1725.2/24	[100]
94	**6U82**	-	DNA/38		1:1	2043.6/38	
95	**7D7V**	17delU1A (riboswitch)	RNA/57	U1 small nuclear ribonucleoprotein A	1:1	918.8/22	[101]
96	**7JTN**	SL1103	DNA ^§^/30	Complement factor B	1:1	844.7/15	[102]
97	7JTQ	SL1102	DNA ^§^/32		1:1	838.2/11	
98	6VUG	38NT2,4-methyl	DNA ^§^/38	HIV-1 reverse transcriptase	1ˆ:1	1874.2/12	[103]
99	**6Z8V**	TBA-3L	DNA ^§^/15	Thrombin (exosite I)	1ˆ:1	605.8/14	[104]
100	6Z8W	TBA-3G	DNA ^§^/15		1ˆ:1	602.4/13	
101	6Z8X	TBA-3Leu	DNA ^§^/15		1ˆ:1	592.2/9	
102	**7F49**	BT-100	RNA ^§^/30	Von Willebrand factor	1:1	1063.3/19	[105]
103	**7MK1**	SL1090	DNA ^§^/41	Antiviral innate immune response receptor RIG-I	1:1	838.8/14	[106]
104	7NTU	NU172	DNA/26	Thrombin (exosite I)	1ˆ:2	591.1/11	[107]
		HD22_27mer	DNA/27	Thrombin (exosite II)		1095.1/18	
105	7OXQ	-	DNA/28 + 21	HIV-1 reverse transcriptase	1ˆ:1ˆ	2459.7/28	[108]
106	**7OZ2**				1ˆ:1ˆ	2421.2/28	
107	7OZ5				1ˆ:1ˆ	2460.2/27	
108	7OZW	38NT2,4-methyl (variant)	DNA ^§^/37		1ˆ:1	1797.8/21	
109	7P15				1ˆ:1	1922.7/20	
110	7LRI	38NT2,4-methyl	DNA ^§^/38		1ˆ:1	2160.9/21	[109]
111	7LRM	38NT2,4-methyl (variant)	DNA ^§^/38		1ˆ:1	2179.3/22	
112	7LRX				1ˆ:1	2219.2/25	
113	7LRY				1ˆ:1	2189.9/22	
114	7LSK	38NT2,4-methyl	DNA ^§^/38		1ˆ:1	2089.1/22	
115	**7SZU**	Pepper	RNA ^§^/67	Fab BL3-6	1ˆ:1	764.7/6	[110]
116	7U0Y				1ˆ:1	774.4/5	
117	**7V5N**	A14#1	DNA/24	Fab fragment of bevacizumab	1ˆ:1	1025.1/19	[111]
118	**7YQ3**	A43	DNA ^§^/28	Insulin receptor	2:1	1272.1/13	[112]
119	**7YQ4**	A62	DNA ^§^/24		2:1	1177.4/8	
120	7YQ5				2:1	1177.4/8	
121	7YQ6				2:2	1188.3/11	
122	7Z24	38NT2,4-methyl (variant)	DNA ^§^/38	HIV-1 reverse transcriptase	1ˆ:1	1683.9/20	[113]
123	7Z29	38NT2,4-methyl	DNA ^§^/38		1ˆ:1	1849.2/20	
124	7Z2D	38NT2,4-methyl (variant)	DNA ^§^/38		1ˆ:1	1538.7/15	
125	7Z2E	38NT2,4-methyl	DNA ^§^/38		1ˆ:1	1578.2/19	
126	7Z2G	38NT2,4-methyl (variant)	DNA ^§^/38		1ˆ:1	2040.0/23	
127	7Z2H	38NT2,4-methyl	DNA ^§^/38		1ˆ:1	1882.7/23	
128	7ZKL	TBA-NNp/DDp	DNA/15	Thrombin (exosite I)	1ˆ:1	576.0/11	[114]
129	**7ZKM**			Thrombin (exosite I)	1ˆ:2	583.1/14	
				Thrombin (exosite II)		595.1/10	
130	7ZKN			Thrombin (exosite I)	1ˆ:2	571.3/12	
				Thrombin (exosite II)		608.5/11	
131	7ZKO			Thrombin (exosite I)	1ˆ:1/1ˆ:2	579.1/9	
				Thrombin (exosite II)		597.0/14	
132	**7ZQS**	tJBA8.1	DNA/51	Transferrin receptor protein 1	2:2	607.6/10	[115]
133	**8D29**	Theophylline aptamer	RNA/34	Fab BL3-6	1ˆ:1	796.9/11	[116]
134	8DK7				1ˆ:1	797.6/8	
135	**8BW5**	M08s-1_41mer	DNA/41	Thrombin (exosite I)	1ˆ:1	585.1/13	[117]
136	**8F3C**	(riboswitch)	RNA/47	DNA-directed RNA polymerase	Large assembly	1661.1/23	[118]
137	8G00				Large assembly	1661.1/23	
138	8G1S				Large assembly	1809.6/25	
139	8G2W				Large assembly	1695.8/19	
140	8G4W				Large assembly	2187.8/37	
141	8G7E				Large assembly	2009.4/22	
142	8G8Z	(riboswitch)	RNA/48		Large assembly	2175.2/23	
143	**8J1Q**	AM032-0	DNA ^§^/80	Spike protein S1 (ACE2-binding site)	1:2	857.5/6	[119]
		AM047-0	DNA ^§^/52	Spike protein S1 (distal site)		748.6/4	
144	**8J26**	AM032-4	DNA ^§^/44	Spike protein S1 (ACE2-binding site)	1:2	867.5/8	
		AM047-6	DNA ^§^/48	Spike protein S1 (distal site)		741.0/4	

^§^ Aptamers containing at least one chemically modified nucleotide. The related PDB entries were manually curated for the analyses. ˆ See Section 2.4 for details. ^‡^ The Holliday junction aptamer is formed by two 17-mer and two 20-mer DNA strands. IA/H-bonds refer to one of the two equivalent protein–aptamer interfaces.

## Supplementary Materials

The following supporting information can be downloaded at: https://www.mdpi.com/article/10.3390/ijms242216318/s1.
