# Supplementary material for: Structural Insights into Protein–Aptamer Recognitions Emerged from Experimental and Computational Studies
Source: Int J Mol Sci. 2023 Nov 14;24(22):16318. doi: 10.3390/ijms242216318 (PMC10671752; doi:10.3390/ijms242216318)
Supplement: Supplementary file 1 [file ijms-24-16318-s001.zip › SupplMat_Sica_p.pdf]

Supplementary Materials for:

Structural Insights into Protein-Aptamer Recognitions Emerged from Experimental and Computational Studies

Romualdo Troisi [1,2,†], Nicole Balasco [3,†], Ida Autiero [2], Luigi Vitagliano [2,*] and Filomena Sica [1,*]

[1] Department of Chemical Sciences, University of Naples Federico II, Naples, Italy; romualdo.troisi@unina.it

[2] Institute of Biostructures and Bioimaging, CNR, Naples, Italy; ida.autiero@cnr.it

[3] Institute of Molecular Biology and Pathology, CNR c/o Department of Chemistry, University of Rome Sapienza, Rome, Italy; nicole.balasco@cnr.it

* Correspondence: luigi.vitagliano@cnr.it (L.V.); filomena.sica@unina.it (F.S.)

† These authors contributed equally to this work.

Supplementary table captions

Table S1. Ensemble of 435 PDB structures identified in the initial PDB search.

Table S2. Ensemble of 144 PDB entries of protein-aptamer complexes.

Table S3. List of the DNA protein-bound aptamers for which similar aptamers were detected in the protein-unbound dataset. The criteria used to assess the similarity were the following: sequence identity > 85% and sequence coverage of the shared fragment > 55% for each counterpart.

Table S4. List of the RNA protein-bound aptamers for which similar aptamers were detected in the protein-unbound dataset. The criteria used to assess the similarity were the following: sequence identity > 85% and sequence coverage of the shared fragment > 55% for each counterpart.

Supplementary figures

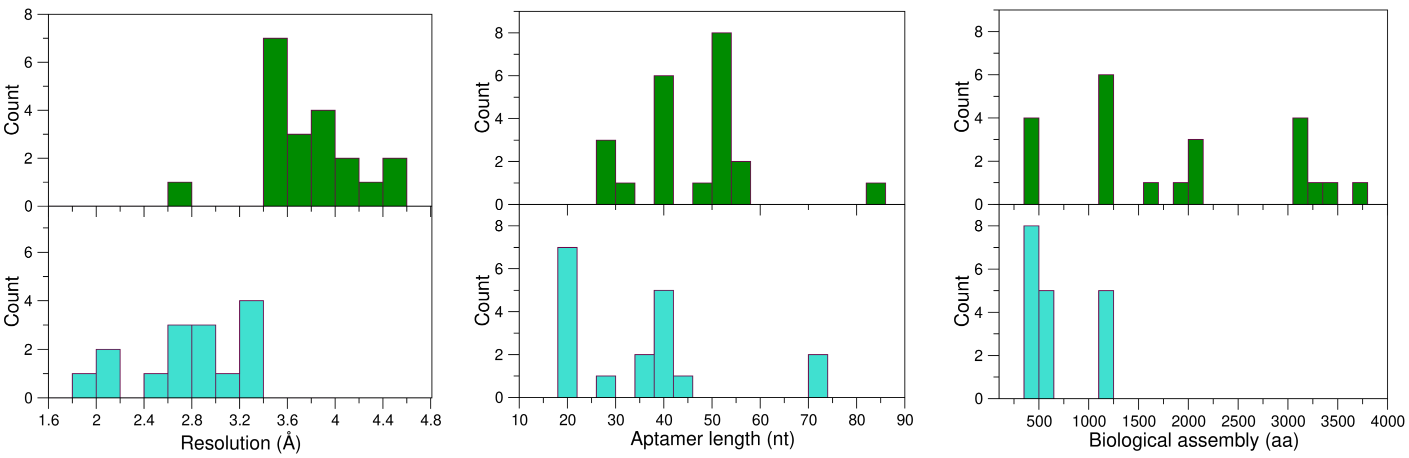

(**a**) (**b**) (**c**)

Figure S1. Distribution in terms of (a) resolution, (b) aptamer length, and (c) protein biological assembly of the structures of protein-aptamer complexes determined since 2022 by X-ray crystallography (15 entries corresponding to 18 interfaces, cyan) and cryo-EM (20 entries corresponding to 22 interfaces, green). Aptamer length and protein biological assembly are reported as the number of nucleotides (nt) and amino acid residues (aa), respectively.

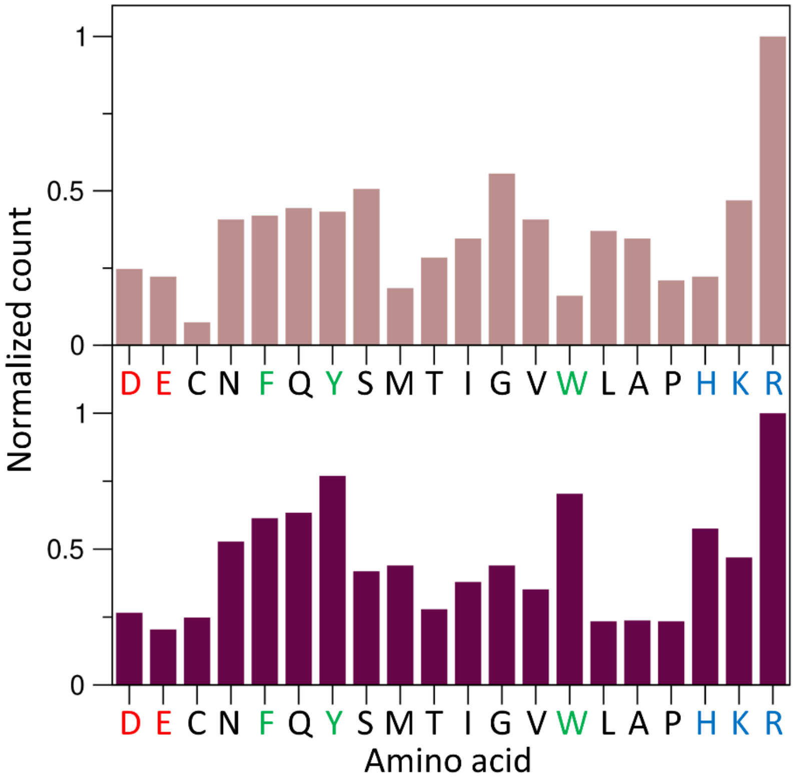

Figure S2. Representativeness of amino acid residues involved in protein interactions with aptamers. Values are normalized for the overall amino acid composition (https://web.expasy.org/protscale/pscale/A.A.composition.html) in the lower panel. Normalized values are given as the ratio of the observed value to the maximum value of the distribution. Aromatic, positively-charged, and negatively-charged residues are in green, blue, and red, respectively. A non-redundant dataset containing 42 PDB entries (PDB IDs 5MSF, 1EXD, 1EXY, 1OOA, 2B63, 3HXO, 3AGV, 3HSB, 3UZS, 3V7E, 4HQU, 2RU7, 3ZH2, 4I7Y, 4M4O, 4NI7, 4PDB, 4R8I, 4WB2, 4YB1, 4ZBN, 5DO4, 5HRT, 5MJV, 5UC6, 6B14, 5VOE, 6CF2, 6RTI, 6SY4, 6TXR, 6U82, 7JTN, 7F49, 7MK1, 7OZ2, 7V5N, 7YQ3, 7ZQS, 8BW5, 8F3C, 8J1Q) has been considered for the analysis. A single entry has been selected for different aptamers interacting with the same protein region.

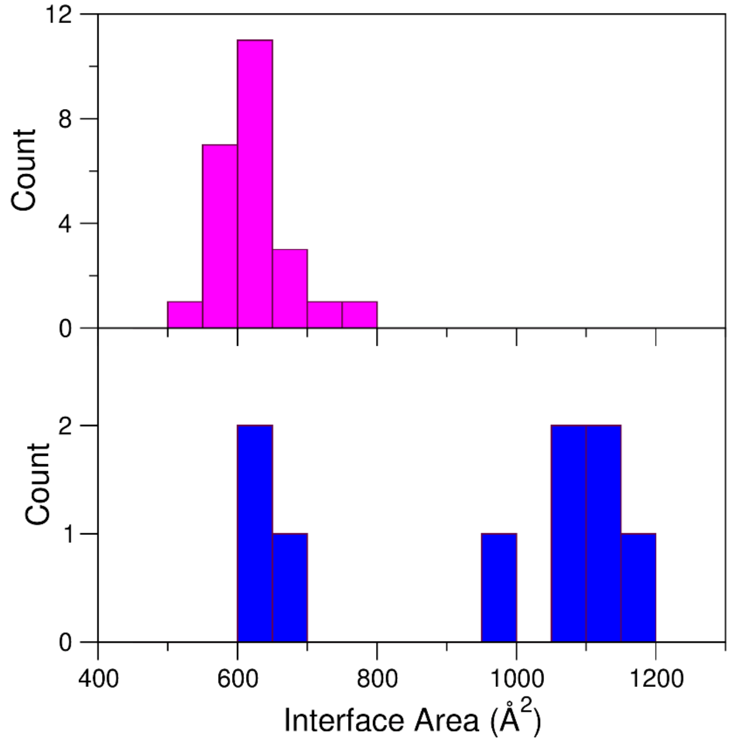

Figure S3. Distribution of the interface areas at exosite I (magenta) and II (blue) of thrombin-aptamer complexes.

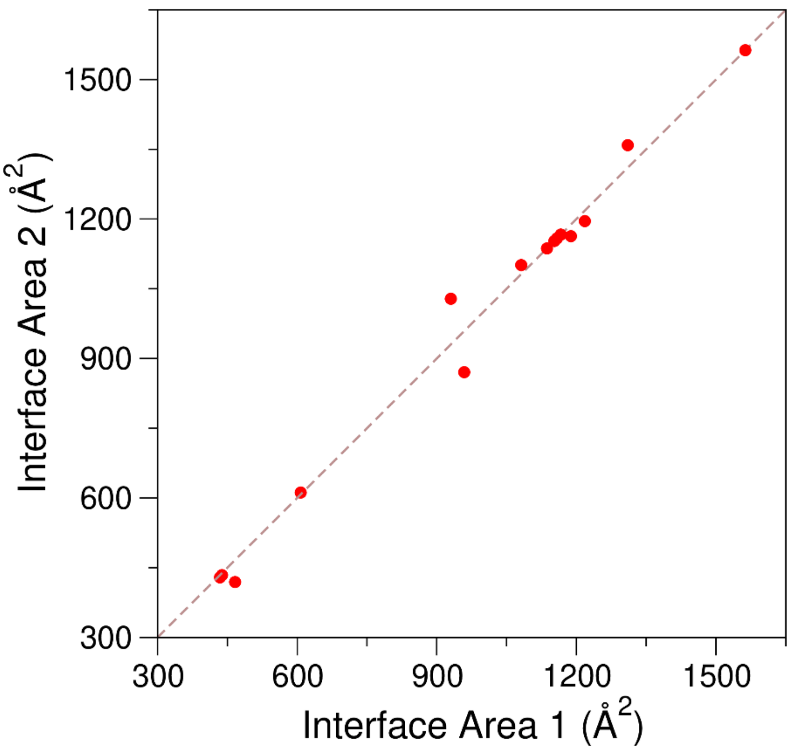

Figure S4. Comparison of the redundant protein-aptamer interface areas present in the same biological assembly of complexes with stoichiometry 2:2, 4:2, and 6:2 (see Table 1).
